# Efficacy of Bilateral Versus Unilateral Transversus Abdominis Plane Blocks in Pain Control After Kidney Transplantation: A Retrospective Review

**DOI:** 10.7759/cureus.105792

**Published:** 2026-03-24

**Authors:** Nathan Goergen, Valerie Shostrom, Arika Hoffman, Heitor J S Medeiros, Rafael Arsky Lombardi

**Affiliations:** 1 Anesthesiology, University of Nebraska Medical Center, Omaha, USA; 2 Surgery, University of Nebraska Medical Center, Omaha, USA; 3 Anesthesiology, Critical Care and Pain Medicine, Massachusetts General Hospital, Boston, USA

**Keywords:** bilateral tap block, bilateral transversus abdominis plane block, kidney transplantation, postoperative opioid consumption, postoperative pain, regional anesthesiology, transversus abdominis plane block, unilateral tap block, unilateral transversus abdominis plane block

## Abstract

Background

Postoperative pain management is a critical component of care following major surgical procedures such as kidney transplantation. Inadequate pain control can increase morbidity, while reliance on systemic opioids may lead to adverse effects. The transversus abdominis plane (TAP) block provides regional analgesia to the anterolateral abdominal wall and has been increasingly used as part of multimodal analgesia strategies. Given conflicting evidence regarding unilateral versus bilateral approaches, this study aimed to determine whether bilateral TAP blocks provide superior early postoperative analgesia compared with unilateral TAP blocks in adult kidney transplant recipients.

Methods

We conducted a retrospective chart review of 83 adult kidney transplant recipients (37 bilateral TAP blocks and 46 unilateral TAP blocks) at a single institution. Postoperative pain scores were assessed using the Numerical Rating Scale (NRS), and opioid consumption was recorded and converted to morphine milligram equivalents (MME). Pain scores and opioid requirements were compared between the bilateral and unilateral TAP block groups.

Results

Bilateral TAP blocks were associated with significantly lower pain scores during the first 12 hours postoperatively for all patients (p=0.0153) compared with unilateral blocks. However, no significant difference was observed in 24-hour pain scores for the overall cohort (p=0.3917). The reduction in pain scores appeared to be driven primarily by male patients, who demonstrated significantly lower pain scores at both 12 hours (p=0.0021) and 24 hours (p=0.0335) when receiving bilateral TAP blocks. In contrast, no statistically significant differences were observed between the groups among female patients at either 12 hours (p=0.7768) or 24 hours (p=0.3545). Total opioid consumption did not differ significantly between bilateral and unilateral TAP block groups for the overall cohort (p=0.9526), male patients (p=0.7024), or female patients (p=0.7511).

Conclusion

Bilateral TAP blocks may provide improved early postoperative analgesia following kidney transplantation, particularly during the first postoperative hours. However, this improvement in pain scores was not associated with a reduction in opioid consumption. Further studies are needed to clarify the clinical significance of these findings and to determine whether specific patient populations may benefit more from bilateral TAP block techniques.

## Introduction

Postoperative pain management is a fundamental component of surgical care. Poorly controlled postoperative pain has been associated with increased morbidity, delayed recovery, prolonged hospitalization, and lower patient satisfaction [1-4]. Although systemic opioids remain widely used for postoperative analgesia, their use is frequently limited by adverse effects such as nausea, vomiting, sedation, and respiratory depression [2]. For this reason, multimodal analgesic strategies that incorporate regional anesthesia techniques have become an important part of modern perioperative care [1].

One regional technique increasingly used for abdominal procedures is the transversus abdominis plane (TAP) block. This block produces somatic analgesia of the anterolateral abdominal wall through the injection of local anesthetic into the fascial plane located between the internal oblique and transversus abdominis muscles [5]. Within this fascial plane run the thoracolumbar nerves that provide sensory innervation to the abdominal wall, typically arising from spinal segments T6 through L1 [5]. Dermatomal coverage with TAP blocks may vary depending on the approach used (e.g., lateral, posterior, or subcostal) and the spread of local anesthetic within the fascial plane, which can influence the extent of sensory blockade achieved. By interrupting these nerve pathways, TAP blocks can reduce the pain associated with abdominal wall incisions, and bilateral blocks may provide broader dermatomal coverage when compared with unilateral approaches.

Kidney transplantation is a major abdominal operation that can result in substantial postoperative discomfort. Effective postoperative analgesia is particularly important in this population because adequate pain control may facilitate early mobilization, improve respiratory function, and enhance overall patient recovery. As a result, TAP blocks have been increasingly incorporated into multimodal analgesic protocols for abdominal procedures and have been associated with improved postoperative comfort and reductions in opioid requirements in several surgical populations [1,2,4,6].

TAP blocks have been studied across a wide range of abdominal operations, including cesarean delivery, hysterectomy, cholecystectomy, colectomy, prostatectomy, and hernia repair [7-13]. These procedures involve somatic pain arising from the abdominal wall, which is supplied by the thoracolumbar nerves traveling through the TAP [5]. In procedures that involve predominantly unilateral incisions, unilateral TAP blocks may be sufficient to provide effective analgesia. For example, laparoscopic cholecystectomy often involves right-sided abdominal incisions, and previous studies have reported that right-sided unilateral TAP blocks may achieve analgesic outcomes comparable to bilateral techniques in this setting [3,5].

Several ultrasound-guided approaches have been described for TAP block placement, including lateral, posterior, and subcostal techniques [4,5]. The oblique subcostal TAP block represents a modification of the traditional subcostal approach and has been proposed as a technique capable of providing broader dermatomal coverage of the upper abdominal wall. This approach allows local anesthetic to spread along the oblique subcostal line, potentially covering nerve distributions from T6 to L1. Ultrasound guidance has become the preferred method for performing TAP blocks because it allows direct visualization of abdominal wall structures, improves injection accuracy, and reduces the likelihood of complications [5,14].

Despite the increasing use of TAP blocks in abdominal surgery, the optimal approach for achieving effective postoperative analgesia remains uncertain. Some investigations have suggested that unilateral TAP blocks may provide analgesia comparable to bilateral blocks in procedures involving unilateral incisions, whereas other studies have reported improved pain control with bilateral blocks [2,3,5,6]. For instance, a study evaluating ureteric shock wave lithotripsy found that unilateral TAP blocks produced analgesic outcomes similar to bilateral blocks [4]. Similarly, in laparoscopic cholecystectomy, unilateral right-sided TAP blocks demonstrated pain control and opioid consumption comparable to bilateral approaches [2].

Given these mixed findings, further research is needed to determine whether unilateral or bilateral TAP blocks provide optimal postoperative analgesia in kidney transplant recipients. While unilateral blocks may reduce total local anesthetic exposure, bilateral blocks may provide broader dermatomal coverage and potentially improve early postoperative pain control.

This study evaluated the efficacy of bilateral versus unilateral TAP blocks for postoperative pain management in adult kidney transplant recipients by examining pain scores and opioid consumption during the early postoperative period. Our objective was to determine whether bilateral TAP blocks provide superior early postoperative analgesia or whether unilateral blocks achieve comparable outcomes in this patient population.

This article was previously presented as a meeting abstract at the American Society of Regional Anesthesia and Pain Medicine Spring Conference (May 1-3, 2025) at Rosen Shingle Creek in Orlando, FL.

## Materials and methods

We conducted a retrospective chart review of adult patients who underwent kidney transplantation at a single institution. This study was reviewed and approved by the Institutional Review Board (IRB) of the University of Nebraska Medical Center (UNMC). Data were collected from patient charts and electronic medical records at the UNMC to evaluate postoperative pain management following a kidney transplant. The study compared the outcomes of patients who received either a unilateral or bilateral TAP block as a part of their postoperative pain management regimen.

Adult patients who underwent kidney transplantation were considered. Patients were included in the study if they received either a unilateral or bilateral TAP block. Patients with an American Society of Anesthesiologists (ASA) score greater than V, those who underwent emergent or redo surgeries, patients with opioid abuse disorder or chronic opioid use, and patients with chronic pain conditions were excluded. The decision to perform a unilateral versus bilateral TAP block was made by the attending anesthesiologist based on clinical judgment and provider preference at the time of surgery. Patients were also excluded if they did not have documentation of a TAP block or had incomplete medical records. After induction of anesthesia, the ultrasound probe was placed along the lateral abdominal wall at the midaxillary line between the costal margin and iliac crest to identify the external oblique, internal oblique, and transversus abdominis muscle layers. A needle was advanced under ultrasound guidance into the fascial plane between the internal oblique and transversus abdominis muscles. Once correct placement was confirmed, 20 mL of 0.25% bupivacaine per side was injected into the TAP with a total of 2-3 mg of dexamethasone added to the local anesthetic solution. Pain scores were recorded by nursing staff into the patient's electronical medical record. 

At our institution, postoperative analgesia following kidney transplantation is typically managed using a multimodal approach that includes scheduled non-opioid and opioid medications. Patients routinely receive acetaminophen (Tylenol) 650-1,000 mg orally every 6 hours as baseline analgesia. Oxycodone 5-10 mg orally every four to six hours as needed is commonly used for breakthrough postoperative pain. Intravenous hydromorphone (0.2-0.5 mg) is administered as rescue analgesia when additional pain control is required.

Data from 83 kidney transplant recipients between January 1, 2021, and January 1, 2024, were reviewed. The primary outcomes were pain scores and postoperative opioid requirements. Pain scores for bilateral (n=37) versus unilateral (n=46) TAP procedures were compared.

Data collection

Pain scores were collected from the electronic medical record at various postoperative time points up to 24 hours. Pain was assessed using the Numerical Rating Scale (NRS) [15] and recorded by bedside nursing staff as part of routine postoperative clinical care. In accordance with standard institutional monitoring practices, pain scores were typically documented approximately every two hours during the early postoperative period. Because this study was retrospective in design, pain assessments were obtained from routine clinical documentation rather than from a standardized research protocol. Data included pain scores at rest and with movement when available.

The total amount of opioid analgesics administered in the first 24 hours post-surgery was recorded, including both intravenous and oral opioid medications. All opioids were converted to morphine milligram equivalents (MME) for analysis. Patient satisfaction scores related to postoperative pain management were collected, as well as demographic variables including age and gender.

Statistical analysis

Statistical analysis was performed using SAS version 9.4 (SAS Inc., Cary, NC, US). A significance level of 0.05 was used for all statistical comparisons, and descriptive statistics including means, standard deviations, medians, and quartiles were calculated for all continuous variables. Frequencies and percentages were calculated for all categorical variables. No formal adjustment for multiple comparisons was performed because the subgroup and time-window analyses were exploratory in nature within this retrospective cohort study. For continuous variables, the t-test was used for normally distributed data and the Mann-Whitney U test was used for non-normally distributed data to compare the bilateral versus unilateral TAP block groups. For categorical variables, the Chi-square test was used to assess group differences. Area under the pain curve (AUC) was calculated for the first 24 hours and 12 hours post-surgery using the trapezoidal rule and compared using the Mann-Whitney U test.

## Results

A total of 83 patients (100%) were included in the final analysis. Among them, 37 patients (44.6%) received bilateral TAP blocks, and 46 patients (55.4%) received unilateral TAP blocks.

Overall, 48 (57.8%) were male patients, and 35 (42.2%) were female patients. Within the bilateral TAP block group, 23 (62.2%) were male patients, and 14 (37.8%) were female patients. Within the unilateral TAP block group, 25 (54.3%) were male patients, and 21 (45.7%) were female patients.

There was no significant difference in 24-hour pain scores between the bilateral TAP group (n=37, 44.6%) and the unilateral TAP group (n=46, 55.4%) when considering all subjects (p=0.3917).

Among male patients, 24-hour pain scores were significantly lower in the bilateral TAP group (n=23, 62.2%) compared with the unilateral group (n=25, 54.3%; p=0.0335). Among female patients, no statistically significant difference was observed between the bilateral group (n=14, 37.8%) and the unilateral group (n=21, 45.7%; p=0.3545).

**Figure 1:**
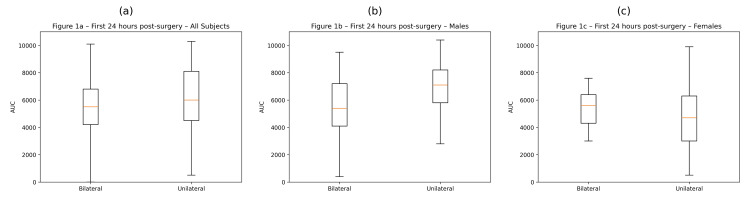
Opioid consumption expressed as AUC during the first 24 hours after kidney transplantation in patients receiving bilateral versus unilateral TAP blocks (a) All subjects (bilateral n=37; unilateral n=46); (b) Male patients (bilateral n=23; unilateral n=25); (c) Female patients (bilateral n=14; unilateral n=21). The central line represents the median, the box represents the interquartile range, and the whiskers represent the range excluding outliers. AUC: Area under the curve; TAP: transverse abdominis plane.

AUC for postoperative opioid consumption was calculated for the first 12 and 24 postoperative hours using the trapezoidal rule, and comparisons between bilateral and unilateral TAP block groups were performed using the Mann-Whitney U test. Boxplot distributions of the 24-hour AUC values are shown in Figures 1a-1c.

Pain scores during the first 12 postoperative hours were significantly lower for all subjects in the bilateral TAP group (n=37, 44.6%) compared with the unilateral TAP group (n=46, 55.4%; p=0.0153). This difference appeared to be largely driven by male patients receiving bilateral TAP blocks (p=0.0021), whereas no statistically significant difference was observed among female patients (p=0.7768).

**Figure 2:**
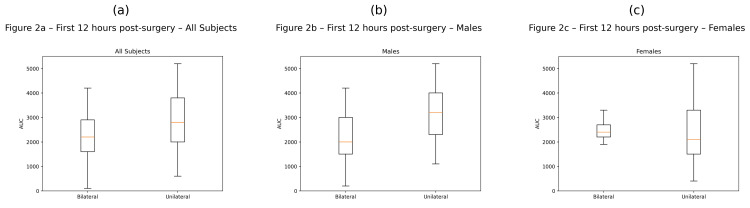
Opioid consumption expressed as AUC during the first 12 hours after kidney transplantation in patients receiving bilateral versus unilateral TAP blocks (a) All subjects (bilateral n=37; unilateral n=46); (b) Male patients (bilateral n=23; unilateral n=25); (c) Female patients (bilateral n=14; unilateral n=21). The central line represents the median, the box represents the interquartile range, and the whiskers represent the range excluding outliers. AUC: Area under the curve; TAP: transverse abdominis plane.

Boxplot distributions of opioid consumption expressed as AUC during the first 12 postoperative hours are presented in Figures 2a-2c.

Additional analyses examining specific postoperative observation windows are summarized in Table 1.

**Table 1:** Pain scores at specific postoperative time windows Median pain scores at different postoperative observation windows following kidney transplantation. n values represent patients with available pain scores within the specified observation window.

Time window	Group	n	Median pain score	p-value
90 minutes at 12 hours	Bilateral	24	4	
	Unilateral	24	5.5	0.0503
	Male Bilateral	15	4	
	Male Unilateral	15	6	0.0288
	Female Bilateral	9	4	
	Female Unilateral	9	4	0.6552
120 minutes at 12 hours	Bilateral	29	4	
	Unilateral	32	5	0.2703
	Male Bilateral	17	4	
	Male Unilateral	19	5	0.0491
	Female Bilateral	12	4	
	Female Unilateral	13	4	0.6797
180 minutes at 24 hours	Bilateral	34	4	
	Unilateral	36	5	0.5425
	Male Bilateral	22	4	
	Male Unilateral	22	5	0.2182
	Female Bilateral	12	5	
	Female Unilateral	14	5	0.5828
240 minutes at 24 hours	Bilateral	34	4	
	Unilateral	37	5	0.5824
	Male Bilateral	22	4	
	Male Unilateral	22	5	0.2182
	Female Bilateral	12	5	
	Female Unilateral	15	5	0.5029

Because pain scores were recorded at variable clinical time points, only patients with documented pain scores within each observation window were included in the corresponding analysis. As a result, the sample sizes reported in Table 1 are smaller than the total cohort sizes of the bilateral (n=37) and unilateral (n=46) groups.

Considering a 90-minute window at 12 hours post-surgery, pain scores were significantly lower in male patients receiving bilateral TAP blocks (p=0.0288), whereas no significant difference was observed for the total cohort (p=0.0503) or female patients (p=0.6552). Within this window, the median pain score for all subjects was 4.00 in the bilateral group (n=24) and 5.50 in the unilateral group (n=24). Among male patients, the median pain score was 4.00 in the bilateral group (n=15) and 6.00 in the unilateral group (n=15). Among female patients, the median pain score was 4.00 in both groups (n=9 in each group).

Within 120 minutes at 12 hours post-surgery, the median pain score for all subjects was 4.00 in the bilateral group (n=29) and 5.00 in the unilateral group (n=32; p=0.2703). Among male patients, the median pain score was 4.00 in the bilateral group (n=17) and 5.00 in the unilateral group (n=19; p=0.0491). Among female patients, the median pain score was 4.00 in both groups (n=12 and n=13, respectively; p=0.6797).

Within a 180-minute window at 24 hours post-surgery, there was no statistically significant difference between groups for all subjects (p=0.5425), with median pain scores of 4.00 in the bilateral group (n=34) and 5.00 in the unilateral group (n=36). Among male patients, the median pain score was 4.00 in the bilateral group (n=22) and 5.00 in the unilateral group (n=22; p=0.2182). Among female patients, the median pain score was 5.00 in both groups (n=12 and n=14; p=0.5828).

Extending the window to 240 minutes at 24 hours post-surgery also showed no statistically significant differences. Median pain scores were 4.00 in the bilateral group (n=34) and 5.00 in the unilateral group (n=37; p=0.5824) for all subjects. Among male patients, median scores were 4.00 (n=22) and 5.00 (n=22; p=0.2182). Among female patients, median scores were 5.00 in both groups (n=12 and n=15) (p=0.5029).

Postoperative opioid consumption data are summarized in Table 2.

**Table 2:** Postoperative opioid consumption (MME) Total postoperative opioid consumption expressed as morphine milligram equivalents (MME). n values represent patients with available opioid consumption data; TAP: transverse abdominis plane.

Group	n	Median MME	p-value
All subjects			
Bilateral TAP	37	22	
Unilateral TAP	45	22	0.9526
Male patients			
Bilateral TAP	23	24	
Unilateral TAP	25	28	0.7024
Female patients			
Bilateral TAP	14	20	
Unilateral TAP	20	18	0.7511

There was no significant difference in total MME between the bilateral and unilateral groups during the first 24 or 48 postoperative hours for the overall cohort or when stratified by sex. The median total MME for the overall cohort was 22.00 in the bilateral group (n=37) and 22.00 in the unilateral group (n=45; p=0.9526).

Among male patients, the median total MME was 24.00 in the bilateral group (n=23) and 28.00 in the unilateral group (n=25; p=0.7024). Among female patients, the median total MME was 20.00 in the bilateral group (n=14) and 18.00 in the unilateral group (n=20; p=0.7511).

## Discussion

The findings of this study suggest that bilateral TAP blocks may provide improved early postoperative analgesia compared with unilateral TAP blocks in adult kidney transplant recipients. Specifically, pain scores during the first 12 postoperative hours were significantly lower among patients receiving bilateral blocks. These findings are consistent with previous studies suggesting that broader dermatomal coverage from bilateral TAP blocks may enhance postoperative analgesia [6].

This effect can be explained by the anatomical course of the thoracolumbar nerves that supply sensory innervation to the anterolateral abdominal wall [5]. These nerves arise from spinal segments T6 through L1 and travel within the fascial plane between the internal oblique and transversus abdominis muscles [4,5,10]. Local anesthetic injected into this fascial plane blocks sensory nerve transmission from abdominal wall incisions. Bilateral TAP blocks may therefore provide more extensive nerve blockade by targeting both sides of the abdominal wall, whereas unilateral blocks affect only the nerves on a single side.

Previous investigations have suggested that the effectiveness of TAP blocks may depend on the spread of local anesthetic within the fascial plane and the resulting dermatomal coverage achieved. In one comparative study evaluating TAP blocks for laparoscopic nephrectomy, bilateral blocks were associated with longer duration of analgesia compared with unilateral blocks [6]. Variability in local anesthetic spread may limit the effectiveness of unilateral blocks, particularly in procedures where multiple dermatomes contribute to postoperative pain [1]. Bilateral blocks may therefore provide more consistent coverage of the abdominal wall nerve supply.

In contrast to the observed differences in early pain scores, this study did not demonstrate a statistically significant reduction in postoperative opioid consumption among patients receiving bilateral TAP blocks. This finding suggests that factors beyond incisional pain may influence opioid use in the postoperative period. Individual variability in pain perception, surgical factors, and the use of additional analgesic modalities may all contribute to opioid requirements following surgery [1]. Similar findings have been reported in other studies evaluating TAP blocks, where improvements in pain scores were not always accompanied by significant reductions in opioid consumption [16].

The surgical context of kidney transplantation may also influence analgesic outcomes. Kidney transplant procedures typically involve lower abdominal incisions and variable degrees of tissue manipulation. While TAP blocks provide effective somatic analgesia of the abdominal wall, postoperative pain following kidney transplantation may also include visceral components that are not fully addressed by this technique, which may partially explain the variability in analgesic outcomes observed across patients [1,17-19]. Additionally, because this investigation was retrospective in design, variations in block timing, injection technique, and surgical practice patterns may have affected the analgesic outcomes observed [6]. This study was also conducted at a single center with a relatively small sample size, and block allocation was based on provider preference rather than randomization, which may introduce selection bias. Furthermore, perioperative analgesic regimens were not strictly standardized, which may have contributed to variability in postoperative analgesic outcomes.

A limitation relates to the availability of pain score documentation at specific postoperative time windows. Because this study relied on retrospective extraction of pain scores recorded in the electronic medical record as part of routine clinical care, not all patients had documented pain scores within every predefined observation window. Pain score availability ranged from 48 of 83 patients (57.8%) in the 90-minute window around 12 hours postoperatively to 71 of 83 patients (85.5%) in the 240-minute window around 24 hours, corresponding to missing data proportions ranging from 42.2% to 14.5% depending on the time window analyzed. Opioid consumption data were available for 82 of 83 patients (98.8%). As a retrospective study, this analysis is subject to several inherent sources of bias. Selection bias may have occurred because the decision to perform unilateral versus bilateral TAP blocks was based on provider preference rather than randomization. Misclassification bias is possible due to reliance on documentation in the electronic medical record, including variability in pain score recording and block documentation. Although recall bias is less relevant given that data were obtained from contemporaneously recorded clinical records rather than patient recall, variability in documentation practices may still affect data accuracy. Additionally, confounding variables such as differences in surgical technique, intraoperative management, and individual patient pain perception were not fully controlled and may have influenced the observed outcomes.

Several areas warrant further investigation. Future studies should examine whether improvements in early postoperative pain scores translate into clinically meaningful outcomes such as shorter hospital stays, improved mobility, or fewer postoperative complications. Additionally, evaluating TAP blocks as a part of multimodal analgesic strategies, incorporating non-opioid medications such as acetaminophen or nonsteroidal anti-inflammatory drugs, may provide further insight into optimizing postoperative pain control.

## Conclusions

Bilateral TAP blocks may provide improved early postoperative analgesia following kidney transplantation compared with unilateral TAP blocks. Although the anatomical rationale supports broader abdominal wall coverage with bilateral blocks, the clinical benefit appears primarily limited to early postoperative pain control and may vary across patient subgroups. These findings highlight the importance of individualized regional anesthesia strategies in kidney transplant recipients. Further prospective studies are needed to better define the clinical role of bilateral TAP blocks and to clarify potential sex-related differences in analgesic response.
